# Assembling strategy to synthesize palladium modified kaolin nanocomposites with different morphologies

**DOI:** 10.1038/srep13763

**Published:** 2015-09-03

**Authors:** Xiaoyu Li, Jing Ouyang, Yonghua Zhou, Huaming Yang

**Affiliations:** 1 Centre for Mineral Materials, School of Minerals Processing and Bioengineering, Central South University, Changsha 410083, China; 2 School of Chemistry and Chemical Engineering, Central South University, Changsha 410083, China

## Abstract

Nanocomposites of aluminosilicate minerals, kaolins (kaolinite and halloysite) with natural different morphologies assembling with palladium (Pd) nanoparticles have been successfully synthesized through strong electrostatic adsorption and chemical bonding after surface modification with 3-aminopropyl triethoxysilane (APTES). Meanwhile, the influence of different morphologies supports on catalytic hydrogenation properties was explored. The surface concentration of amino groups on the kaolins was related to the morphology and surface nature. Electronmicroscopy revealed that the monodisperse Pd nanoparticles were uniformly deposited onto the surface of kaolins, ranging in diameter from 0.5 nm to 5.5 nm. The functional groups could not only improve the dispersion of kaolins with different morphologies in solution, but also enhance the interaction between Pd precursors and kaolins, thus preventing small Pd nanoparticles from agglomerating and leading to high activity for the catalytic hydrogenation of styrene. Pd-FK@APTES was more active compared to other samples. Selecting the kaolin morphology with a different surface nature allows the selective surface modification of a larger fraction of the reactive facets on which the active sites can be enriched and tuned. This desirable surface coordination of catalytically active atoms could substantially improve catalytic activity.

Nanocomposites can synergize properties between phases because of the nanoscale dispersion of the nanoparticles on the matrix and the interface can elicit new functional properties^1^. Recently, nanoscale Pd particles have drawn particular attention due to their highly effective catalytic^2^ and electronic properties^3^. However, the synthesized Pd nanoparticles with high surface area are easily agglomerated, which greatly limits their application. In order to enhance the performance, Pd catalysts have been anchored on different supports so as to avoid the particles aggregation and movement, such as mesoporous materials (SBA-15^4^, MCM-41^5^, porous alumina^6^ and carbon nanotubes^7^) and layered materials^8^ and so on. The majority of researches are focused on nanoparticles anchoring on a single support or morphology-controlled synthesis of samples with the same chemical composition, and there has been very little research into the production of composites containing the more supports which possess the natural different morphologies but the similar chemical composition. Also, the quantitative data on the comparison of catalytic hydrogenation activities of different catalyst morphologies have been lacking.

As we all know, the properties of nanocomposites are closely associated with their size, morphology, phase and structure^9^. Therefore, it is essential to study the formation of nanoparticles on the supports with different morphologies, whose surface property maybe specifically tuned. Kandite group minerals, kaolins (kaolinite and halloysite) are such supports with different morphologies but the similar chemical composition, and attract more and more attention. In general, clay minerals naturally possess various morphologies, including fiber, sheets, scrolls, rods, and tubes. The morphological diversity is created in the process of the formation of clay minerals in nature. Kaolins are important aluminosilicate minerals of the kandite group, which also includes dickite and nacrite, and so on^10^. Kaolins can exist in different morphologies (nanoflakes^11^, nanotubes^12^ and nanorods^13^), which are natural and abundant nanomaterials for versatile applications. Kaolinite is a clay mineral with 1:1 dioctahedral layered structure whose layer consists of AlO_2_(OH)_4_ octahedral sheets and SiO_4_ tetrahedral sheets^14^,^15^, stacked like a deck of cards. A similar structure is observed for naturally occurring nanotubular halloysite^16^,^17^, which differs from kaolinite in the intercalated water. The external surface is composed of siloxane (Si−O−Si) groups, whereas the internal surface consists of a gibbsite-like array of aluminol (Al−OH) groups^18^. The different chemical constitutions determine the negatively charged outer surface and positively charged inner lumen within a certain pH range^19^,^20^. For kaolins, the different morphologies determine the different surface charge distributions, thus leading to the different surface natures^21^,^22^, which allows the selective surface modification and the adjustable Pd size, which further affects the catalytic performance.

In this paper, we focus on the different morphologies and surface nature of kaolins, which were used as effective supports for anchoring Pd nanoparticles, and investigate the influence of different morphologies on their catalytic hydrogenation performance. Pd nanoparticles dispersed on the surface of modified kaolins with different morphologies without combination can make full use of the active sites, and improve their properties. Selecting the kaolins morphology with a different surface nature allows the selective surface modification of the reactive facets on which the active sites may be specifically enriched and tuned.

## Results

**Figure 1:**
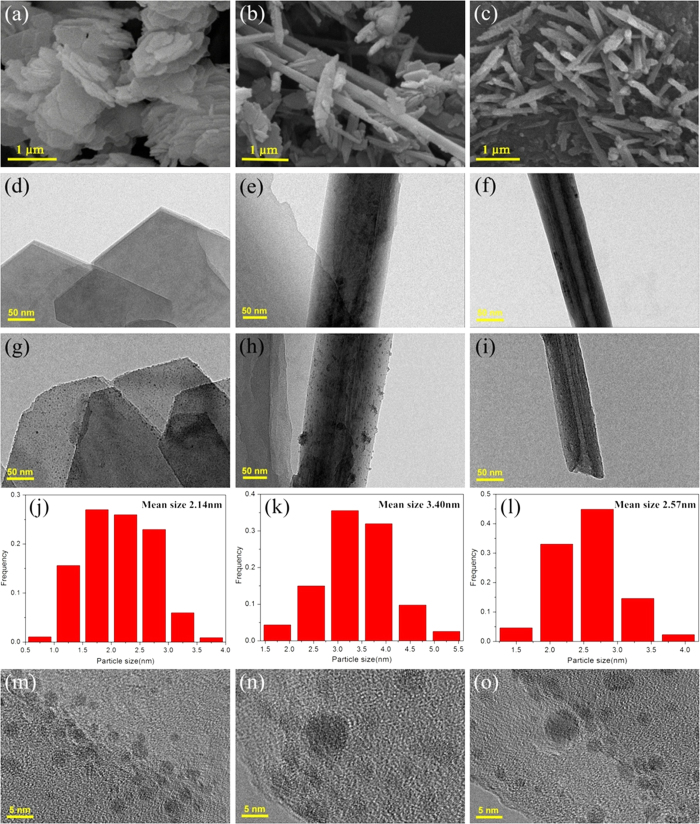
Morphology of the samples. SEM and TEM images of kaolins with different morphologies: (**a**,**d**) flake-like, (**b**,**e**) rod-like, and (**c**,**f**) tube-like. TEM and HRTEM images of Pd-kaolins@APTES, and the corresponding histogram of Pd clusters diameters: (**g**,**j**,**m**) flake-like, (**h**,**k**,**n**) rod-like, (**i**,**l**,**o**) tube-like.

**Figure 2:**
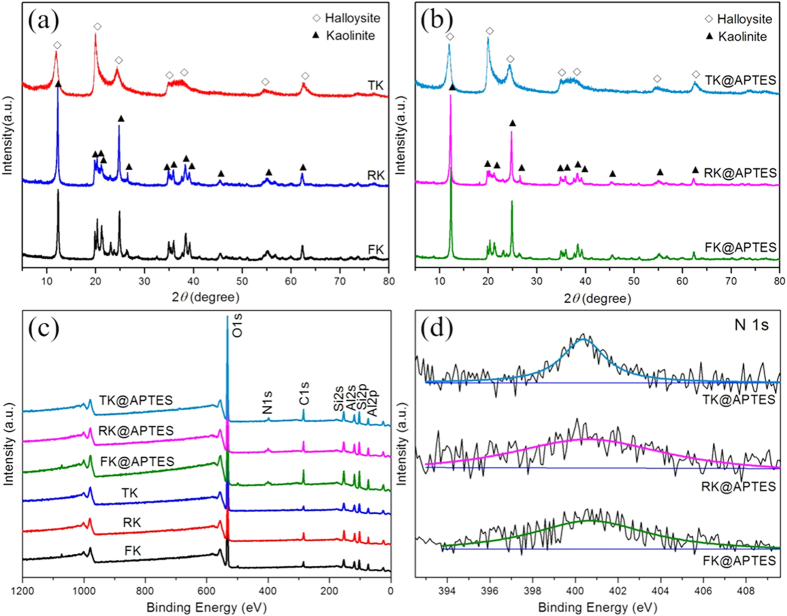
Crystallization and spectra of the samples. XRD patterns of (**a**) kaolins with different morphologies, (**b**) surface modified kaolins. (**c**) XPS survey spectra of kaolins and surface modified kaolins. (**d**) N 1s fitted XPS spectra of surface modified kaolins.

Kandite group, as pervasive aluminosilicate minerals in the earth’s crust, can naturally exist in different morphologies, which are natural and abundant nanomaterials for versatile applications. Typical SEM analysis indicated that the kaolins all possessed smooth surface without contamination and different morphologies (Fig. 1a–c). The XRD patterns of the kaolins showed the reflections which were in good agreement with kaolinite and halloysite structure, respectively (Fig. 2a). The phase of flake-like and rod-like kaolin was kaolinite, while the tube-like kaolin was halloysite. Actually, kaolinite and halloysite are important clay minerals of the kandite group, which also include dickite and nacrite and possess different morphologies. Kaolinite nanoflakes possessed an irregular angular shape and the length of particle was in the range of microns (Fig. 1a,d). Nanorod-like kaolinites with smooth surface (Fig. 1b,e) were mostly 2–5 *μ*m in length and 0.1–0.3 *μ*m in diameter with a length to width ratio of about 20:1. Meanwhile, some platelets with the irregular shapes were also observed in Fig. 1b. Nanotube-like kaolins (halloysites) predominately consist of cylindrical nanotubes 50–150 nm in diameter and 1–2 *μ*m in length (Fig. 1c). Actually, halloysite is a naturally occurring hydrated polymorph of kaolinite with tubular morphology (Fig. 1f), and has a similar structure and composition, but the unit layers are separated by an additional monolayer of water molecules^23^. As a result, hydrated halloysite has a basal spacing d (001) of 10 Å, which is ~3 Å larger than that of kaolinite. The intercalated water is weakly held and can be readily and irreversibly removed^24^. Furthermore, little change was observed after surface modification with APTES (Fig. 2b). However, according to the XPS survey spectra (Fig. 2c) and the high-resolution N 1s fitted XPS spectra (Fig. 2d) of kaolins and surface modified kaolins, the existence of nitrogen and carbon species in the functionalized kaolins confirmed the success of the surface modification reaction performed on kaolins, which was attributed to −NH^3+^ groups with the binding energy around 400.6 eV^25^,^26^.

**Figure 3:**
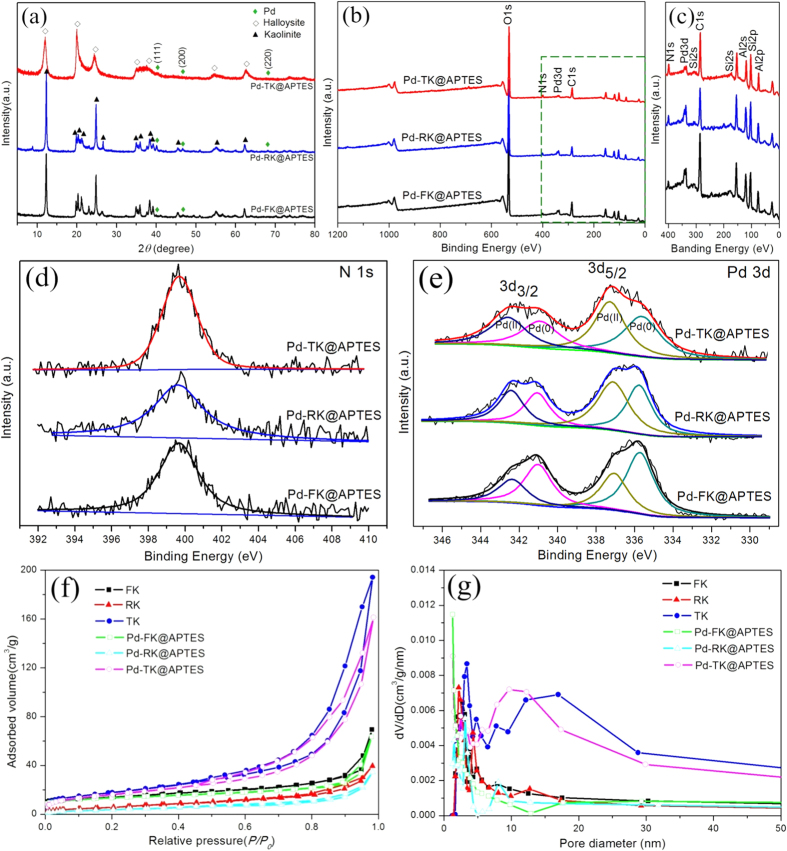
Phase, spectra and texture of the samples. (**a**) XRD patterns, (**b**) XPS survey spectra, (**c**) magnified part over the range of 410–0 eV, (**d**) N 1s and (**e**) Pd 3d fitted XPS spectra of Pd-kaolins@APTES, (**f**) Nitrogen adsorption–desorption isotherms and (**g**) corresponding BJH pore size distribution of kaolins and Pd-kaolins@APTES.

After the surface modification, APTES were successfully grafted on the surface of kaolins, and the Pd-kaolins@APTES nanocomposites were prepared by means of strong electrostatic adsorption and chemical immobilization. The XRD patterns of the as-synthesized Pd-kaolins@APTES showed that the reflections at 2*θ* of about 40.1°, 46.7° and 68.1° (Fig. 3a), which corresponded to the crystal planes of the (111), (200) and (220) of face-centered cubic (fcc) Pd (JCPDS standard card No.65-2867, space group: F*m*3*m*)^27^, respectively. It is obvious that the spacing of the kaolins barely changed after surface modification and introduction of the reduced Pd nanoparticles, which indicated that the structure of kaolins was maintained and Pd nanoparticles were not intercalated in kaolins but present at the exterior of the kaolins layer. Although the patterns showed weak crystalline peaks for Pd nanoparticles, probably because of the low Pd content and/or small particle size^28^, which suggested that the Pd atoms were well-dispersed on the surface of kaolins. The XPS survey spectra and magnified part over the range of 410–0 eV of Pd-kaolins@APTES materials (Fig. 3b,c), which provided definite evidence for the presence of Pd. Moreover, the high-resolution N 1s fitted XPS spectra exhibited a lower binding energy of about 399.7 eV, may be ascribed to amide groups (Fig. 3d)^25^,^29^, which could provide quantitative information about the functional groups based on the peak area. Pd-TK@APTES grafted the highest amount of functional groups than Pd-FK@APTES and Pd-RK@APTES, which was also consistent with the ICP-AES results (Table 1). The different degree of functionalization was related to the various morphologies and unique surface nature of kaolins. Meanwhile, the intensity of N1s fitted peaks for Pd-kaolins@APTES was higher than that of surface modified kaolins (Fig. 2d), and shifted to the lower binding energies, demonstrating the strong interaction between Pd nanoparticles and −NH_2_ groups^30^.

For kaolins, the FTIR spectra were similar with each other (Figure S1), but the absorption bands between 3800 and 3500 cm^−1^ attributed to –OH stretching were sensitive to structural disorder^31^. Because the unit layers of halloysite are separated by an additional monolayer of water molecules (3450 cm^−1^)^12^,^23^, its crystallinity and order go down compared to kaolinite (flake-like and rod-like). As a result, an overall decrease in the intensity of the halloysite was observed. Furthermore, with the increase of the structure order degree, kaolinites exhibited fairly narrow bands across FTIR spectra. Meanwhile, the absorption bands number was increased and the intensity was stronger than that of halloysite. For flake-like kaolinite, the absorption bands observed at around 3694 and 3620 cm^−1^ were ascribed to the stretching of the interlayer hydroxyl groups pointing almost perpendicularly to the *c*-direction^32^,^33^, and the band at 3658 cm^−1^ was assigned to the inner surface –OH out-of-phase stretching vibration^34^. The other bands at 3740, 1115, 1033 and 915 cm^−1^ were assigned to the Si–OH stretching vibration, apical Si–O stretching vibration, the Si–O stretching vibration and the vibration of inner Al–OH groups, respectively. The bands at 793, 753 and 690 cm^−1^ were due to the vibration of O–Al–OH. The bands at 545, 470 and 429 cm^−1^ were attributed to the vibration of Si–O–Al^35^,^36^. There was little change in the band positions of rod-like kaolinite compared with flake-like kaolinite, only the overall intensity were slightly reduced. It was observed that most band positions of the Pd-kaolins@APTES did not change compared to that of kaolins, suggesting that the basic crystal structures of kaolins and Pd-kaolins@APTES remained constant. However, the vibration bands at 2930 and 2862 cm^−1^ were newly observed, which could be attributed to C−H asymmetric and symmetric stretching vibrations^37^,^38^, respectively, thereby confirming the anchorage of the propyl groups on the clays surface. The band at 1465 cm^−1^ was confirmed by the presence of −CH_2_ deformation^38^. A vibrational band at around 1655 cm^−1^ was responsible for the presence of an imine group formed by the oxidation of anamino group^39^. The peak at 1304 cm^−1^ can be assigned the stretching vibration of C–N band. The above results may indicate that silane coupling agent, the sole source for C–H and N–H, had been successfully grafted onto the kaolins surface. New bands were observed, indicating strong interactions between APTES and the kaolins surfaces. It was noted that the intensity of new formed bands for Pd-FK@APTES was stronger than those of the other Pd-kaolins@APTES. However, the N 1s fitted XPS spectra (Fig. 3d) and ICP-AES results showed that Pd-TK@APTES grafted the highest amount of functional groups than Pd-FK@APTES and Pd-RK@APTES. It implied that the surface modification was accomplished on both the external surface and inner lumen of the halloysite nanotubes. Furthermore, it was reported that the chemical modification of phyllosilicates by grafting with silanes occurs mainly on the hydroxyl groups of the surface of the clay^33^. It was confirmed by the hydroxyl intensity decrease of Pd-kaolins@APTES compared with kaolins. Therefore, different morphologies kaolins possessed different amount surface hydroxyl groups resulted in the different degree of functionalization under the same experimental condition and further affected the loading amount of Pd nanoparticles. Meanwhile, in order to verify the state of Pd on the surface of kaolins, the high-resolution fitted XPS measurements of Pd 3d were carried out (Fig. 3e). Pd-kaolins@APTES all contained two different Pd surface species. Pd 3d_5/2_ and Pd 3d_3/2_ peaks around 335.7 and 341 eV were ascribed to the metallic Pd only, whereas peaks at 337.1 and 342.4 eV corresponded to Pd 3d_5/2_ and Pd 3d_3/2_ peaks of oxidized Pd–O species, respectively, which suggested that the reduction process led to only a partial reduction of Pd(II) to Pd(0) for Pd/kaolins@APTES.

In addition, the adsorption–desorption isotherms of kaolins and Pd-kaolins@APTES and Barrett–Joyner–Halenda (BJH) pore size distribution were shown in Fig. 3f,g, and the textural parameters calculated from the corresponding isotherms were summarized in Table 1. The BET specific surface area (*S*_BET_) and pore volume (*V*_pores_) of the functionalized kaolins were evidently smaller than those of kaolins, which could be attributed to the surface modification and deposition of the Pd nanoparticles. However, N_2_ adsorption–desorption isotherms of kaolins and corresponding Pd-kaolins@APTES were very similar (Fig. 3f), which indicated that the pore structures of kaolins were not damaged heavily after the functionalization and Pd nanoparticles loading, which was in agreement with the XRD results. Particularly, the isotherms of halloysite and Pd-TK@APTES were more distinct with other samples, and exhibited the type II with *H3* hysteresis loops according to IUPAC classification^23^,^40^, thereby suggesting that halloysite nanotubes possess a larger amount of macropores leading to higher specific surface area, bigger pore volume and stronger absorption ability than other kaolinites. The pore size distribution for halloysite nanotubes showed two distinct peaks around 3.4 and 14.7 nm, indicating two primary populations of pores. The population of about 15 nm can be readily identified as the lumen of the halloysite, while the 3.4 nm population may correspond to the spaces between particles in bundles, or lumen with partially closed openings^40^. Pd-TK@APTES exhibited a major pore population centered at 10.3 nm, which was slightly narrowed relative to that of halloysite. The pore sizes of functionalized kaolinites were also decreased compared with original kaolinites. Due to the specific nanotube morphology of halloysite, during the course of the experiment by vacuum cycling, partial silanes and palladium solution were inhaled into halloysite lumen. After *in-situ* reduction, Pd nanoparticles were loaded on both the external surface and inner lumen of the halloysite nanotubes. It also confirmed the ICP-AES results that the Pd loading in Pd-TK@ASPTES was higher than that in Pd-FK@ASPTES and Pd-RK@ASPTES.

## Discussion

During the synthesis process, the degree of functionalization was different and could be adjusted due to the various morphologies and unique surface nature of kaolins. It is very important to study the surface nature of the support. The kaolins particles are composed of sheets of tetrahedrally coordinated silica and sheets of octahedrally coordinated alumina and these sheets occur stacked upon one another, but they form different morphologies, leading to different surface nature. Therefore, the surface charge distributions for different morphologies kaolins were discussed and depicted in Figure S2. Under the reaction conditions employed, kaolins with different morphologies possess a net negative charge. Studies of surface charge distributions for clay sols indicate that clay particles carry a net negative charge. The origins of this charge on the clay lattice are believed to be isomorphic substitution, lattice imperfections, broken bonds at the edges of the particles, and exposed structural hydroxyls^41^. Van Olphen^42^ suggested that the principal source of the observed negative charge on the clay particles was isomorphic substitution of cations of lower charge for cations of higher charge within the lattice (e.g., Al^3+^ for Si^4+^ in tetrahedral sheets and Fe^2+^ or Mg^2+^ for Al^3+^ in the octahedral sheet. Meanwhile, the edges of clay particles carry positive charges because of protonation of various atoms exposed at the edges. The dissociation of structural hydroxyl groups is another possible source of negative charge. Additionally, kaolins possessing different morphologies result in different surface charge distribution. The silicate planes of kaolins possessing different morphologies all carry a negative charge, attributed to the partial isomorphous replacement of Si^4+^ by Al^3+^ 43. Kaolinites (nanoflakes and nanorods) have a negative surface charge and a weak positive rim charge. Although the chemical composition of halloysite is similar to that of kaolinites, the chemistry of the outermost surface of halloysite can be associated with that of SiO_2_ and the inner cylindrical surface Al(OH)_2_ entities due to its nanotube-like morphology^43^. As a result, the inner wall of the tube is mainly positively charged, while the outer surface has a weakly negative charge^44^. The different surface charge distributions for kaolins can affect the modified degree, and then affect the deposition of Pd nanoparticles onto the clays surface. The surface modifications improve the wettability of kaolins in solution, and enhance the interaction between palladium precursors and kaolins supports. Thus, there were more negative charges on the surface of kaolins that was more beneficial to anchor the palladium precursors.

The overall synthetic routes of the Pd-kaolins@APTES nanocomposites were illustrated (Figure S3). The kaolin surface possessed a number of negatively charged sites, which could attract APTES complex through electrostatic interaction, and then the negatively charged palladium precursor were deposited onto the surface of kaolins by means of an electrostatic interaction with the positively charged APTES pre-adsorbed onto the surface of the clays. The chemical *in-situ* reduction process reduced Pd(II) species to generate Pd nanoparticles. And then, the Pd-kaolins@APTES nanocomposites were obtained by centrifugation (see experimental section for details).

TEM clearly showed the tubular morphology of halloysite with an external diameter of 75 nm, an inner diameter of 16 nm and a wall thickness of about 28 nm (Fig. 1f), all kaolins with different morphology displayed smooth surfaces (Fig. 1d–f). Through the surface modification, Pd nanoparticles were uniform spherical particles deposited onto the surface of kaolins and presented predominantly as isolated particles (Fig. 1g–i). Moreover, the corresponding EDS spectra of the as-prepared Pd-kaolins@APTES powders showed the presence of Pd, Al, O and Si, which confirmed the existence of Pd nanoparticles on the kaolins (Figure S4). Figure 1j–l displayed histograms of the Pd particle size distribution (0.5–5.5 nm) that were derived from the TEM images of Pd-kaolins@APTES based on 600 Pd clusters’ measurements (Fig. 1g–i). It is well known that the morphologies and surface properties of kaolins usually affect the Pd dispersion and particle size and Pd nanoparticles size distribution could be controlled. The amino groups not only could improve the dispersion of kaolins in the solution, but also could prevent Pd nanoparticles from agglomerating. Relative to other nanocomposites, the Pd nanoparticles exhibited small populations of clumping with a broad size distribution for Pd-RK@APTES composites and the mean particles size was much larger (3.40 nm) than other samples. The agglomeration of Pd nanoparticles was due to the small amount of functional groups on the surface of rod-like kaolinite, which was confirmed by the XPS analysis (Figs 2 and 3), leading to the weak interactions between Pd precursors and rod-like kaolinite. As a result, Pd nanoparticles agglomeration occurred at the reduction process. In contrast, Pd nanoparticles on Pd-FK@APTES were much more uniform and smaller than other samples. The average size of Pd nanoprarticles was approximately 2.14 nm (Fig. 1g–j). Moreover, the HRTEM image further proved the homogeneity of Pd nanoparticles supported on flake-like kaolinite (Fig. 1m).

However, the ICP-AES measurement results confirmed that the Pd loading in Pd-TK@ASPTES was higher than that in other samples (0.94 wt% vs. 0.76 wt% and 0.61 wt%) in Table 1, which could be likely ascribed to the different morphologies and amounts of surface functional groups on these kaolins. Halloysite has a predominantly hollow tubular structure in the submicron range and the inner cylindrical pores are approximately 15–20 nm in diameter, so halloysite has a larger surface area of 64.91 m^2^/g than other kaolinites. The halloysite lumen produced a large capillary force in the solutions, which helped in loading APTES within the tubes. As a result, APTES was absorbed onto both the inner and outer surface of tubular halloysite, which was consistent with previous XPS (Figs 2d and 3d) and ICP-AES results (Table 1). Therefore, it is feasible that a portion of small Pd nanoparticles may have been deposited inside the tubes rather than on the outer-surface. This would also account for the higher amount of nanoparticles evident on the halloysite composite surface relative to the corresponding kaolinite materials in ICP-AES results (Table 1).

TEM also confirmed the presence of Pd nanoparticles inside the cylindrical pores of halloysite showed small nanoparticles incorporated within the halloysite tubes (Fig. 1i), particularly near the ends/openings of these tubes. The HRTEM images depicted clearly visible lattice fringes that evince the formation of crystalline Pd nanoparticles (Fig. 1m–o). The periodicity of the lattice is approximately 0.224 nm, which coincided with the (111) *d*-spacing of the Pd crystal. Since the morphology and surface charge distribution of the kaolins is different from each other, thus leading to the selective modification of APTES on the surface of kaolins. As a result, the active sites can be adjusted, and then affect the particle size, loading amount and dispersion of Pd particles. Correspondingly, by selecting the morphology of kaolins, the resultant property may be specifically tuned.

**Figure 4:**
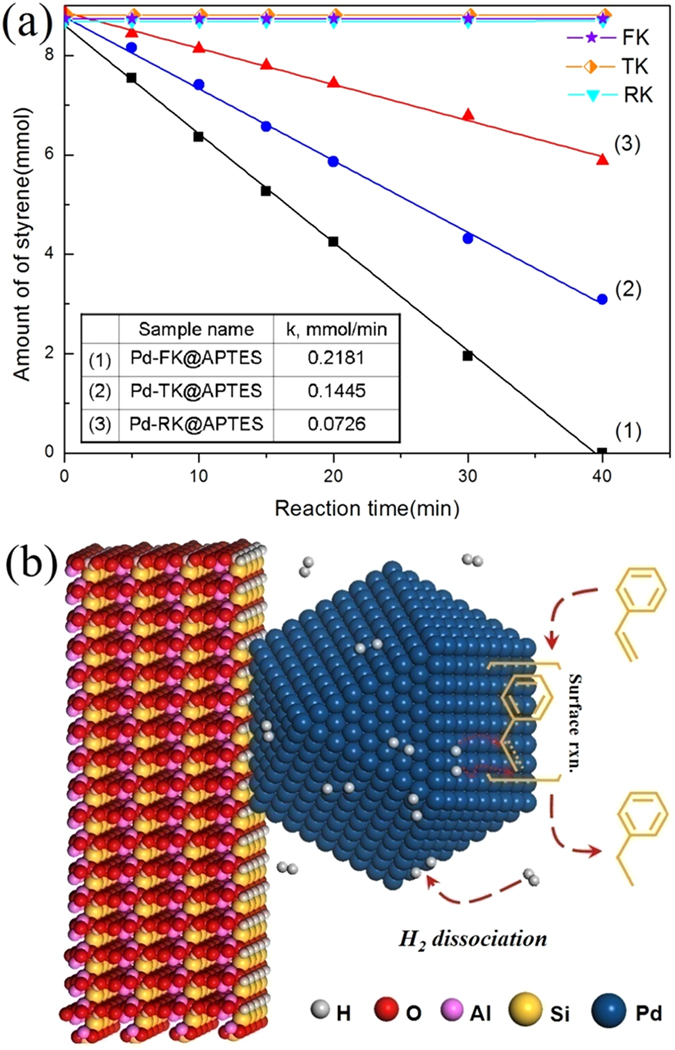
Catalytic activity and proposed reaction mechanism of the samples. (**a**) Styrene hydrogenation reaction curves of Pd-kaolins@APTES, (**b**) Atomic-level schematic diagram for catalytic hydrogenation of styrene.

To evaluate the catalytic activity of Pd nanoparticles supported on kaolins with different morphologies, the styrene hydrogenation was chosen as a probe reaction. In the catalytic experiments, the hydrogenation of styrene led ultimately to the formation of ethylbenzene. The hydrogenation curves for the three catalysts were displayed in Fig. 4a. It is well known that this reaction can be catalyzed by Pd nanoparticles at room temperature^45^ and catalytic hydrogenation essentially refers to chemical reactions on the surface of the catalyst^46^. Moreover, the atomic-level schematic diagram for styrene hydrogenation offered molecular insight into the progress of catalytic hydrogenation under mild conditions (Fig. 4b). Hydrogen molecules were absorbed on the surface of Pd nanoparticles and then dissociated into hydrogen atoms. After that, the active hydrogen atoms attacked styrene moleculars adsorbed on the Pd surface. As a result, styrene was converted to ethylbenzene. It appeared likely that this process was strongly affected by Pd dispersion or the particle size. Extensive studies over the past few decades have demonstrated that the size and shape of catalyst particles on the nanometer scale profoundly affect its reaction performance^47^. Little change existed for the kaolins (Fig. 4a), which indicated that the clay itself had no catalytic activity. The catalytic hydrogenation activities of kaolins assembling with Pd nanoparticles without the surface modification (Pd-FK, Pd-RK and Pd-TK) exhibited lower catalytic activity than those of Pd-kaolins@APTES (Figure S5). This turned out that kaolins possessed various morphologies and plentiful hydroxyl groups on its surface, after surface modification could prevent small Pd nanoparticles from aggregation and movement, thus leading to highly effective catalytic efficiency for the catalytic hydrogenation of styrene. Pd nanoparticles anchored on different morphology kaolins exhibited different styrene catalytic hydrogenation efficiency. The styrene conversion efficiency reached about 100%, 64% and 32% in 40 min for Pd-FK@APTES, Pd-TK@APTES and Pd-RK@APTES, respectively. It was reported that styrene hydrogenation reaction intrinsic kinetics coincides with zeroth-order reaction law^48^, therefore, the rate of the conversion of styrene keeps constant in the entire reaction process. For each Pd-kaolins@APTES sample, the initial reaction rate was calculated from the slope of the straight line to give the activity of the catalyst in terms of (mmol styrene)/min and the results were listed in Fig. 4a. According to the linear regression analysis of the catalytic data, it is obvious that Pd-FK@APTES was a most active catalyst for the styrene hydrogenation under mild conditions. In general, catalytic activity is related to the loading amount and particle size of catalysts. TEM images in previous text prove that the smaller Pd nanoparticles were more easily formed and more highly dispersed on the flake-like kaolinite than other samples, which led to a greatly increased activity for styrene hydrogenation. The reaction finished within 40 min. The catalytic activity of kaolins could be neglected, indicating that Pd nanoparticles were the active sites in this reaction. Pd-RK@APTES showed a lower catalytic activity than Pd-FK@APTES because of larger particle size and lower loading amount of Pd nanoparticles on RK@APTES with respect to FK@APTES. Meanwhile, the Pd-TK@APTES also exhibited lower catalytic activity than Pd-FK@APTES. The loading amount of Pd nanoparticles on TK@APTES was higher than FK@APTES, but not all Pd nanoparticles were active sites for the hydrogenation reaction. Because catalytic hydrogenation of styrene reaction is a surface reaction, it is the outer-surface sites which take part in the hydrogenation of styrene, and the inner surface sites do not display any significant contribution to this reaction. Moreover, the larger particle size also accounted for the lower catalytic activity. The catalytic results revealed that the exposed surface sites, rather than the total number of surface sites, were the catalytically active centers for the hydrogenation of styrene to ethylbenzene. Compared with the reported Pd nanoparticles supported on the other clays, it was shown that the as-synthesized nanocatalysts were more effective for the catalytic hydrogenation of styrene to ethylbenzene.

In summary, kaolins possessing the natural different morphologies but the similar chemical composition were functionalized successfully with APTES and the Pd nanoparticles were well assembled on the modified kaolins through strong electrostatic adsorption and chemical bonding. Meanwhile, the efficient catalytic hydrogenation performance has been compared under mild conditions. The results implied that the functional groups could enhance the interaction between Pd precursorsand kaolins supports to prevent Pd nanoparticles from agglomerating. Morevoer, the samples in the series of Pd-kaolins@APTES catalysts had different morphologies supports with various surface charge distribution, leading to the different degree of surface functionalization, and then affecting the active sites, Pd dispersion, loading amount and mean particle size on the kaolins. Relative to other composites, Pd-FK@APTES composites showed higher activity for the catalytic hydrogenation of styrene, which was attributed to the much smaller size, higher amount of active sites and more uniform distribution of Pd nanoparticles on flake-like kaolinite. Furthermore, Pd-TK@APTES composites possessed a greater concentration of Pd nanoparticles but lower activity than Pd-FK@APTES. This was proposed to be due to the structural difference of the clays. Thus, selecting the kaolin morphology with a different surface nature allows the selective surface modification, and makes the the desirable surface coordination of catalytically active sites could be tuned and substantially improve the catalytic activity. Therefore, our insight into the comparison of different morphologies of kaolins with different surface nature to catalytic hydrogenation performance could be a reference function to the similar investigation. We also believe that the as-synthesized nanocomposites could have interesting potential application in the catalytic fields.

## Methods

### Material preparation

The surfaces of kaolins with different morphologies were modified using commercially available silane reagent. Kaolins were soaked into 25 mL anhydrous toluene and sonicated for 30 min, and then added the APTES solution (1 mL APTES dissolving in 25 mL anhydrous toluene). The mixture was further refluxed at 120 °C for 24 h under constant stirring. After stirring, the solid products were separated from solution and washed by centrifugation with anhydrous toluene repeatedly before drying at 80 °C. The surface modified kaolins labeled as FK@APTES, RK@APTES and TK@APTES were respectively soaked into a 0.1 wt% Na_2_PdCl_4_ solution for 1 h and then heated to 120 °C for 2 h under vacuum. Subsequently, the samples were reduced by NaBH_4_ solution, the color of the samples changed from yellowish-brown to dark-brown, suggesting the formation of metallic palladium nanoparticles on the surface. Finally, the samples were thoroughly washed and dried at 60 °C in a vacuum oven and labeled as Pd-FK@APTES, Pd-RK@APTES and Pd-TK@APTES. For comparison, kaolins assembled with Pd nanoparticles without the surface modification were also obtained via a similar process and labeled as Pd-FK, Pd-RK and Pd-TK.

### Characterization

The structural characteristics of the resulting materials were determined by powder X-ray diffraction (XRD), which were recorded on a D/MAX2550VB + X-ray diffractometer using Cu K*α* radiation (*λ* = 0.15406 nm) at a scanning rate of 0.02°/s with a voltage of 40 kV and 40 mA. The morphologies of samples were observed using a scanning electron microscopy (SEM, FEI Quanta-200) with an accelerating voltage of 5 kV, a transmission electron microscopy (TEM, JEOL JEM-2100F) and high-resolution transmission electron microscopy (HRTEM, JEOL JEM-3010) operating at 200 kV. The as-synthesized samples for TEM analysis were dispersed in ethanol by ultrasound and a drop of each solution was deposited on a Cu grid coated by a holed carbon film and dried in air. Pd clusters were identified by energy-dispersive X-ray analysis (EDX) (equipped with the TEM instrument). The Pd cluster size distributions were determined by counting the sizes of 600 clusters on TEM images taken from different places. The existence of functional groups and their chemical nature were studied by Fourier transform infrared (FTIR). The spectra of the samples over the range of 4000–400 cm^−1^ were recorded on a Nicolet Nexus 670 FTIR spectrophotometer using KBr pellets, and the mixture was pressed into a pellet for IR measurement. X-ray photoelectron spectroscopy (XPS) has been performed to determine the elemental composition, identify the surface groups, the chemical state of the atoms, and the relative abundance in the different synthesized samples. The measurements were taken using a spectrometer (ESCALAB 250; Thermo Fisher Scientific) with a monochromatic Al K*α* source at 1486.6 eV, a voltage of 15 kV, and an emission current of 10 mA. Besides, quantitative determination of functional groups was performed using an inductively coupled plasma-atomic emission spectrometer (ICP-AES, IRIS advantage 1000). The loading level of NH_2_ sourced from APTES was calculated by the nitrogen content of the analytic results. The textural properties of samples were determined by N_2_ porosimetry. The N_2_ adsorption isotherms were recorded at 77 K and analyzed using an ASAP 2020 Surface Area analyzer (Micromeritics Co. Ltd.). The catalytic hydrogenation of styrene reaction was monitored by gas chromatographic (SHIMADZU 2010, column RTX-5 with inner diameter 0.25 mm, film depth 0.25 *μ*m and length 30 m) analysis of the solution withdrawn from the reactor for a given time.

### Catalytic activity evaluation

The catalytic hydrogenation of styrene to ethylbenzene was carried out in a 150 mL glass batch reactor equipped with a magnetic stirrer at 25 °C at atmospheric pressure. For each experiment, 100 mg sample was firstly suspended in 100 mL ethanol together with 0.1 mL *n*-decane (used as the internal standard) and pretreated in H_2_ flow (40 mL/min) at 25 °C for 1 h. Then 1 mL styrene was added into the solution in H_2_ flow under stirring at atmospheric pressure. 1 mL solution was withdrawn and filtered for gas chromatographic test for a given time.

## Additional Information

**How to cite this article**: Li, X. *et al.* Assembling strategy to synthesize palladium modified kaolin nanocomposites with different morphologies. *Sci. Rep.* **5**, 13763; doi: 10.1038/srep13763 (2015).

## Supplementary Material

Supplementary Information

## Figures and Tables

**Table 1:** Summary of characterization results.

Samples	ICP-AES results: Mass concentration (%)		Textural characteristics of samples	
	N 1s	Pd 3d	*S*_BET_ (m^2^/g)	*V*_pores_ (cm^3^/g)
Pd-FK@APTES	7.36	0.76	24.338	0.079
Pd-RK@APTES	7.04	0.61	15.815	0.054
Pd-TK@APTES	13.24	0.94	60.598	0.250
FK			26.876	0.087
RK			26.548	0.061
TK			64.908	0.300
