# Supplementary material for: Assembling strategy to synthesize palladium modified kaolin nanocomposites with different morphologies
Source: Sci Rep. 2015 Sep 3;5:13763. doi: 10.1038/srep13763 (PMC4558717; doi:10.1038/srep13763)
Supplement: Supplementary Information [file srep13763-s1.pdf]

Supporting Information

Assembling strategy to synthesize palladium modified kaolin nanocomposites with different morphologies

Xiaoyu Li, Jing Ouyang, Yonghua Zhou, Huaming Yang*

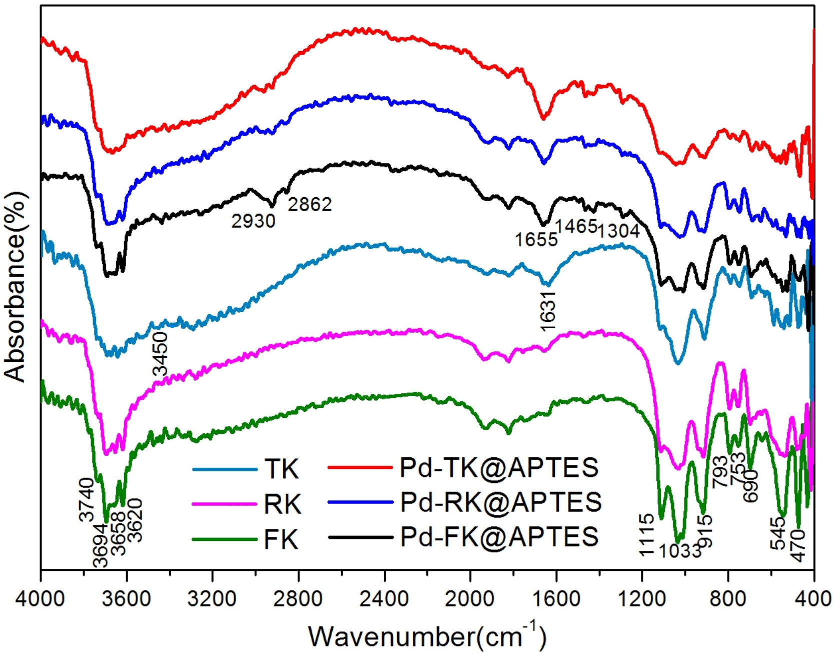

Figure S1. FTIR spectra of kaolins with different morphologies and Pd-kaolins@APTES.

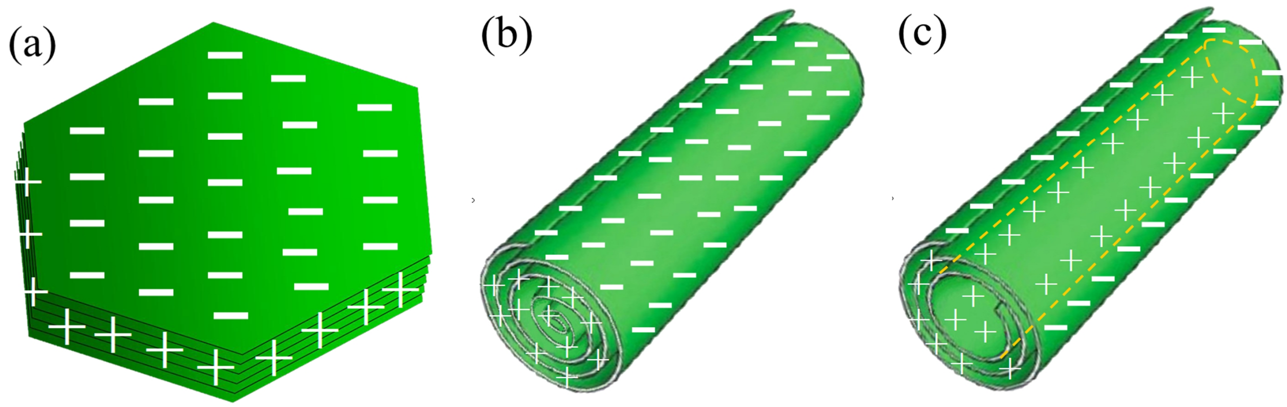

Figure S2. Schematic view of surface charge distributions of kaolins with different morphologies (a) flake-like, (b) rod-like, (c) tube-like.

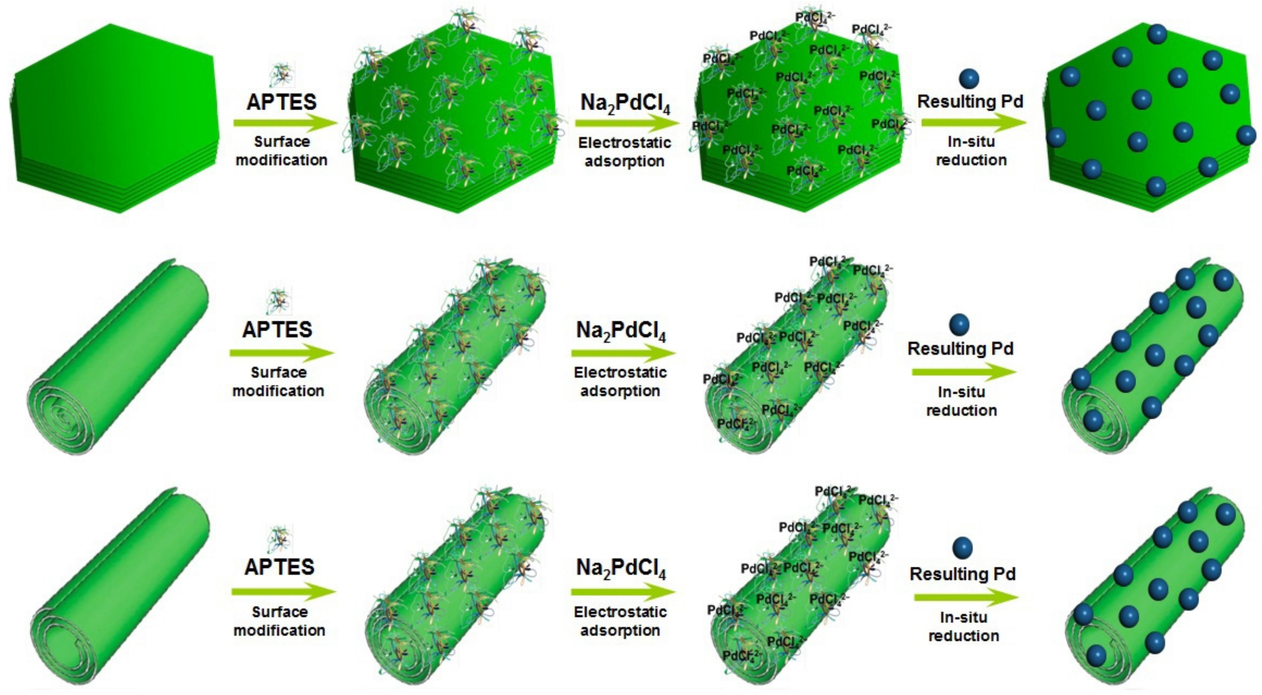

Figure S3. Schematic view of the process of anchoring Pd nanoparticles on kaolins with different morphologies.

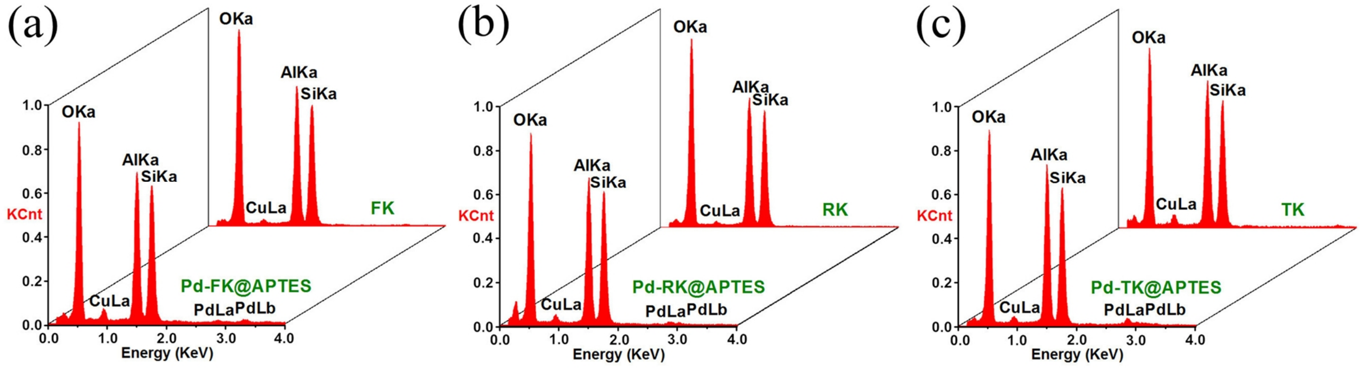

Figure S4. EDS spectra of (a) FK and Pd-FK@APTES, (b) RK and Pd-RK@APTES, (c) TK and Pd-TK@APTES.

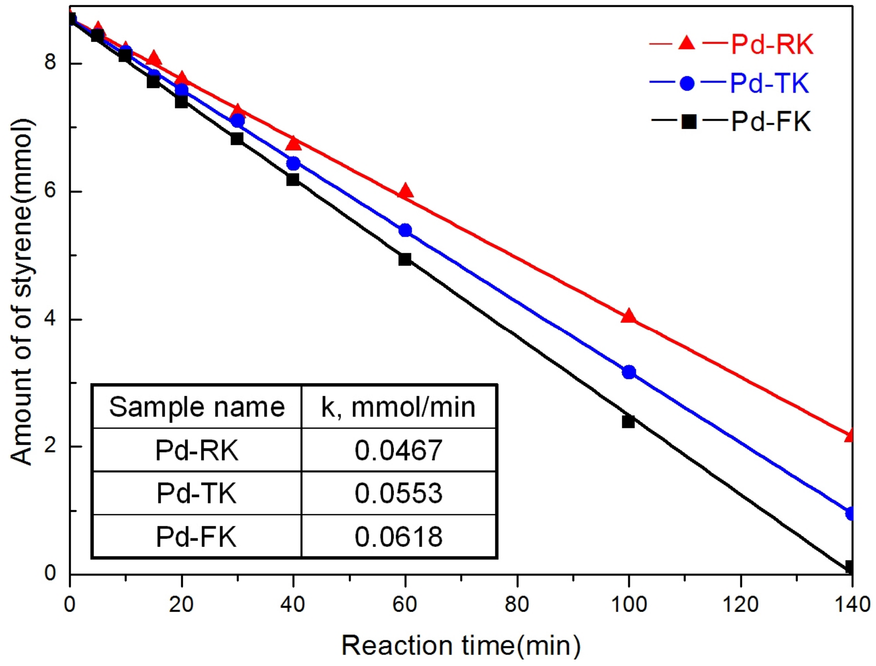

Figure S5. Styrene hydrogenation reaction curves of Pd-RK, Pd-TK and Pd-FK.
